# Bioreactance-Based Noninvasive Fluid Responsiveness and Cardiac Output Monitoring: A Pilot Study in Patients with Aneurysmal Subarachnoid Hemorrhage and Literature Review

**DOI:** 10.1155/2020/2748181

**Published:** 2020-09-15

**Authors:** Sanjeev Sivakumar, Christos Lazaridis

**Affiliations:** ^1^Department of Neurology, Prisma Health-Upstate, University of South Carolina, Greenville, SC, USA; ^2^Departments of Neurology and Neurosurgery, The University of Chicago, Chicago, IL, USA

## Abstract

Management of volume status, arterial blood pressure, and cardiac output are core elements in approaching the patients with aneurysmal subarachnoid hemorrhage (SAH). For the prevention and treatment of delayed cerebral ischemia (DCI), euvolemia is advocated and caution is made towards the avoidance of hypervolemia. Induced hypertension and cardiac output augmentation are the mainstays of medical management during active DCI, whereas the older triple-H paradigm has fallen out of favor due to lack of demonstrable physiological or clinical benefits and serious concern for adverse effects such as pulmonary edema and multiorgan system dysfunction. Furthermore, insight into clinical hemodynamics of patients with SAH becomes salient when one considers the frequently associated cardiac and pulmonary manifestations of the disease such as SAH-associated cardiomyopathy and neurogenic pulmonary edema. In terms of fluid and volume targets, less attention has been paid to dynamic markers of fluid responsiveness despite the well-established, in the general critical care literature, superiority of these as compared to traditionally used static markers such as central venous pressure (CVP). Based on this literature and sound pathophysiologic reasoning, reliance on static markers (such as CVP) is unjustified when one attempts to assess strategies augmenting stroke volume (SV), arterial blood pressure, and oxygen delivery. There are several options for continuous bedside cardiorespiratory monitoring and optimization of SAH patients. We, here, review a noninvasive monitoring technique based on thoracic bioreactance and focusing on continuous cardiac output and fluid responsiveness markers.

## 1. Introduction

Delayed cerebral ischemia (DCI), after aneurysmal subarachnoid hemorrhage (SAH), affects approximately 30% of patients [1]. Hypovolemia has been classically associated with DCI, and standard clinical protocols and guidelines advocate the avoidance of and, instead, maintenance of euvolemia [2, 3]. Assessment of “volume status,” however, tends to be more nuanced both in conceptual and practical terms, as it will be elaborated further. Particularly, the common practice of relying on hourly or daily fluid balances has been shown to be poorly indicative of effective circulating blood volume (which is the variable of interest as a fundamental determinant of cardiac output (CO) and its ability to vary in response to changing metabolic demands) [4, 5]. This often translates into highly subjective, clinician, and institution-specific fluid management targets [6, 7]. It also commonly results in the administration of excessive fluid volumes subjecting patients to the risks of systemic complications such as hypoxemic respiratory failure, kidney injury, and counterproductively decreased cerebral oxygen delivery and worse neurological outcome [8–11]. Monitoring and manipulation of effective circulating volume and CO is not only relevant for the prevention and treatment of DCI but also becomes important in understanding and managing SAH-related cardiorespiratory complications such as stress cardiomyopathy and neurogenic pulmonary edema. The presence of these complications in a patient with high risk, or who is experiencing DCI, poses a potentially great clinical challenge and calls for advanced hemodynamic monitoring [12–14]. In this manuscript, we aim to review a technology based on thoracic bioreactance that provides noninvasive, continuous bedside measurements and monitoring of cardiac index (CI) and dynamic markers of fluid responsiveness, report our pilot study on bioreactance-based fluid responsiveness and cardiac output monitoring in ten patients with aneurysmal subarachnoid hemorrhage, and provide a narrative review of the literature.

### 1.1. Thoracic Bioreactance-Based Hemodynamic Monitoring

**Figure 1:**
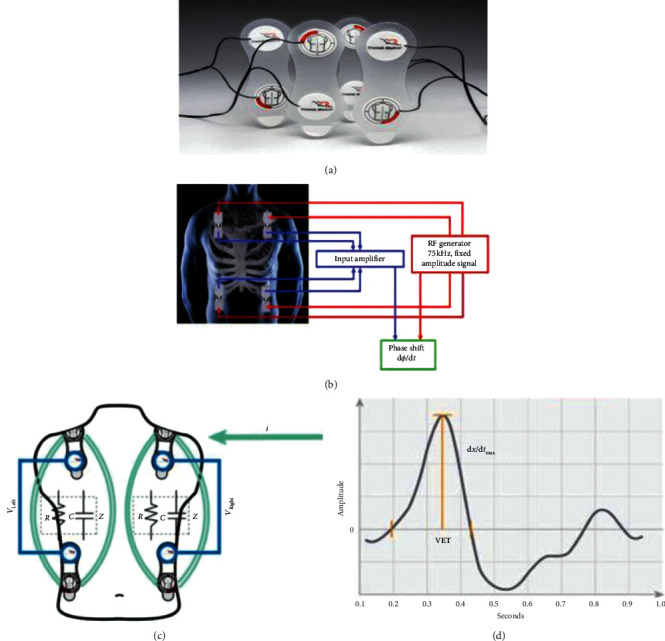
The NICOM system and its connection to the body. (a) Four double-electrode stickers are placed around the thorax. (b and c) A high-frequency current is passed between the 2 outer electrodes, and the resulting voltages are recorded between the 2 inner electrodes. The relative phase shift (*ɸ*) and rate of change of phase (d*ɸ*/d*t*) between these signals are determined and used in the calculations of stroke volume (SV). RF radio frequency (d) schematic representations of aortic flow as a function of time underlying the basic principle for estimation of SV from changes in relative phase shifts (d*x*/d*t*_max_) and ventricular ejection time (VET).

Employing thoracic electrical bioimpedance/reactance for deriving hemodynamic variables is based on the assumption that changes in intrathoracic blood volume, during the cardiac cycle, induce changes in the electrical conductivity of the thorax that are mainly related to changes in aortic volume (in what follows, we will concentrate on bioreactance; bioimpedance-based methodologies face a number of technical and pathophysiologic limitations making them less suitable for intensive care unit (ICU) environments, e.g., extraneous electrical interference). Conductivity changes are detected by skin electrodes assessing the difference between input and output voltage after applying a low-amplitude high-frequency current to the thorax (Figure 1). Bioreactance assesses pulsatile flow-induced frequency modulations and phase shifts in voltage across the thorax. A further physiologic assumption proportionally relates voltage phase shift and stroke volume (estimated using the ventricular ejection time and the slope of the change in aortic volume, see Figure 1). The bioreactance signal-processing method yields a 100-fold reduction in the impact of extraneous electrical fields on CO estimates [15–17]. A noninvasive CO measurement signal is determined separately from each side of the body, and the final noninvasive CO measurement signal is obtained as an average. The noninvasive bioreactance CO monitoring system (NICOM; Cheetah Medical, Newton Center, MA) has been used as a reference standard (for bioreactance-derived CO) as it has shown acceptable agreement with other CO monitoring systems using arterial waveform, [18] and pulse-contour analysis, [19] as well as compared with continuous pulmonary artery thermodilution in an experimental setting, in mixed ICU patients, and patients undergoing off-pump coronary artery bypass surgery [17, 20, 21].

### 1.2. Pilot Experience in Aneurysmal Subarachnoid Hemorrhage

We pilot-trialed the Starling-SV monitor in 10 prospective adult patients with SAH (supplementary material Table 1 provides a summary of patient characteristics; all patients were recruited in the period 2016-2017). This trial aimed to study a prospective convenience sample of SAH patients using an entirely noninvasive device with the goals of assessing user-friendliness of the device and to collect observational data in relation to cardiac function and fluid responsiveness trajectories during ICU stay. All clinical management decisions were independent to study conductance and dictated according to institutional protocol; Starling-SV data were not included, nor available, for any kind of clinical decision making. The study was approved with a waiver of consent by the Baylor College of Medicine IRB (protocol H- 38318) and by the Institute for Clinical and Translational Research at Baylor Saint Luke's Medical Center.

Consecutive patients were recruited and monitored after explanation of the monitoring device and the pilot trial. Data were collected from day 1 until discharge from the neurointensive care unit. Daily assessments of fluid responsiveness (every 12 hours if feasible) were performed. Hemodynamic variables collected included cardiac index, blood pressure, central venous pressure (CVP) (as available), and stroke volume variation (SVV), as well as transcranial Doppler (TCD) mean flow velocities (as available). Volume management variables followed included daily I/O status, hourly urine output (UOP), and type and frequency of fluids and vasoactive medication support provided. Transthoracic echocardiography (TTE) was also performed as part of standard examination in SAH patients on admission and when clinically indicated by changing hemodynamics. Fluid responsiveness was tested by passive leg raise testing (PLR); PLR is a well-described positional maneuver that mobilizes fluid from the lower body towards the central circulation and is one of the recommended methods of testing fluid responsiveness in critically ill patients. If PLR could not be performed, then a fluid challenge of 250 cc of normal saline was used. Whenever fluid boluses were administered, the volume was incorporated and accounted for in the overall fluid management of these patients by adjustment of the total fluid rate provided (by institutional practice, all patients with SAH received maintenance fluids during their ICU stay).

The median age of the 10 participants was 57 years (supplementary material Table 1). The median APACHE II score was 5.5. The median WFNS grade was 1, and the median GCS was 15. Two patients required mechanical ventilation upon presentation. Five patients presented with a modified Fischer scale of 4. Locations of aneurysms were the anterior communicating artery (4), posterior communicating artery (4), middle cerebral artery (1), and superior cerebellar artery (1). Majority of aneurysms were secured by endovascular coiling (8), while two patients were treated with surgical clipping. No patient had a history of heart failure. The median left ventricular TTE ejection fraction was 60%. Four patients developed hyponatremia, and 4 patients developed hydrocephalus during their ICU course. One patient had radiographic evidence of vasospasm, which required endovascular treatment. Delayed cerebral ischemia was defined by a two-point decrease in the Glasgow Coma Scale or NIH stroke scale or the development of a new focal neurological deficit not explained by other factors and the development of a new infarct on neuroimaging; DCI occurred in two patients, who developed a new infarct on the follow-up CT scan. The median ICU length of stay was 12 days. The median modified rankin scale at discharge was 3. Seven patients had a net negative fluid balance during the course of their hospitalization. In terms of TCD data, they were available on 6 patients. Four patients met criteria for ultrasonographic vasospasm (MFV > 120 cm/sec AND LR > 3); 1 patient had features of hyperemia (MFV > 120 cm/sec and LR < 3); one patient had a normal TCD MCA MFV. Summary statistics in terms of mean CI, SV, and PLR responses is reported in Table 3 of the supplementary material: cardiac index, stroke volume, and fluid responsiveness. Overall, the group studied had normal cardiac function (based on both bioreactance and TTE data) and preserved fluid responsiveness throughout their course, as expected by the large majority of patients with low-grade SAH. We did not have any failures in obtaining continuous cardiac index, nor technical mishaps with either device connectivity or performing passive leg raise testing. One patient out of 10 refused to undergo some of the PLRs. There were no complications or patient concerns related to applying the device, and clinical staff rated it as easy to implement.

## 2. Discussion

Induction of hemodynamic augmentation to improve cerebral perfusion is a mainstay response to DCI despite the lack of randomized trials for this intervention [3]. Accumulating literature has shifted the focus from triple-H therapy to the maintenance of euvolemia and induced hypertension [22]. If the goal is to optimize forward flow towards the brain, then preload assessment and fluid responsiveness ought to be routinely interrogated in patients with SAH. This can often be challenging in the absence of a single clinical gold standard. Nevertheless, the decision to administer fluids should not be taken lightly in view of accruing evidence on the harmful consequences of positive fluid balance in neurologic and general ICU patients [23–25] and, specifically, patients with SAH [9, 26–28]. The frequently employed static preload filling-pressure markers such as CVP and pulmonary artery occlusion pressure have been shown to correlate poorly with ventricular filling volumes and fluid responsiveness in healthy volunteers, [29] and critically ill patients [30, 31]. Furthermore, an expert panel specifically and strongly recommended against the use of CVP alone as a target or safety endpoint for guiding fluid therapy in neurocritically ill patients [32]. Contrary to this recommendation, we recently found that the use of static markers and the clinical assessment of volume status remain the most commonly employed variables by intensivists treating acute brain injury patients [33]. Another common practice is the one following daily fluid balances (DFB) to ascertain euvolemia. Different authors have invalidated this practice by documenting poor correlation of DFB with direct measurements of circulating blood volume in patients with SAH using integrated pulse spectrophotometry and pulse dye densitometry [4, 34]. Physiologically speaking, there can be no other reason for volume expansion than to augment stroke volume. This requires biventricular preload dependence, i.e., on Frank–Starling terms, it requires both ventricles to be operating on the ascending part of their performance curves.

Bedside prediction of the ventricular performance is dramatically improved by the use of dynamic variations in the arterial waveform due to heart-lung interactions during positive pressure mechanical ventilation [35–38]. The end-expiratory occlusion test (EEO) together with ultrasonography is a static hemodynamic monitoring method, wherein interruption to the respiratory cycle at the end of expiration averts the expected cyclical changes in venous return and cardiac output. The EEO cannot be used in nonintubated patients and in patients who interrupt a 15 sec inspiratory hold. Pulse-contour analysis has been commonly used to detect the effects of EEO on CO and its validation. Studies employing bioreactance-based CO measurement while employing EEO are lacking. Stroke volume variation (SVV) and pulse pressure variation have been shown to be the most accurate dynamic predictors. Nevertheless, there are a number of limitations in the use of these dynamic markers that may confer false predictions [39]. These include small tidal volumes (TV < 8 ml/Kg), spontaneous breathing activity, open chest conditions, and atrial fibrillation (where the observed beat-to-beat SVV is secondary to altered cardiac filling times and cannot be used as a surrogate of ventricular responses to varying intrathoracic pressures). In the presence of such limitations, assessment of fluid responsiveness can be reliably assessed via the simple PLR bedside test. This involves elevation of the patient's legs to 45° (from an initial semirecumbent position) leading to an autotransfusion of volume pooled in the lower extremities and pelvic veins. Detection of increased SV with this maneuver has been shown to be an accurate predictor of fluid responsiveness (as mentioned above, rapid detection via continuous monitoring of SV is needed since the change may be significant yet transient and short-lived) [40–42].

The CO measured by bioreactance has been shown to be highly correlated with that measured by pulse-contour analysis and thermodilution. The most frequently used analytic method for evaluating CO monitoring devices is the Bland–Altman method of plotting bias against mean CO and by determining the limits of agreement (LOA) [43]. The percentage error, calculated as the ratio of 2 standard deviations of the bias (LOA) to the mean CO, is considered clinically acceptable if below 30% [44]. Lamia et al. performed cross comparisons of trending accuracies of continuous CO measurements between the bolus thermodilution pulmonary artery catheter (PAC), arterial pulse-contour analysis (LiDCOplus™, FloTrac™, and PiCCOplus™), and bioreactance (NICOM™) [45]. Repetitive simultaneous estimates of CO obtained from the abovementioned devices were compared in 21 cardiac surgery patients during the first 2 hours after surgery. Mean and absolute values for CO across the devices were compared; dynamic changes in CO, estimated by each device, showed good cross correlations. Although all devices recorded similar mean CO values, which dynamically changed in similar directions (induced by therapeutic interventions), they demonstrated markedly different bias and precision values relative to each other, i.e., should not be considered interchangeable. Squarra et al. compared CO derived from NICOM with PAC in 110 patients after cardiac surgery [16]. The reported bias was +0.16 L/min; the LOA was ±1.04 L/min with a relative error of 9%. NICOM was able to track changes in CO accurately with a better precision compared to thermodilution. In a study of 70 patients in intensive care units, Raval et al. reported a bias of −0.09 L/min and LOA ±2.4 L/min [17]. NICOM closely tracked changes in thermodilution CO. In a study of simultaneous CO measurements using NICOM, thermodilution, and Fick methods among patients undergoing right-heart catheterization for pulmonary hypertension, the CO measured by using the NICOM system was significantly more precise than thermodilution (3.6% ± 1.7% vs. 9.9% ± 5.7%, *p* < 0.001) [46]. A systematic review and meta-analysis compared CO measured by bolus thermodilution with commercially available noninvasive technologies including pulse wave transit time, noninvasive pulse-contour analysis, thoracic electrical bioimpedance/bioreactance, and CO_2_ rebreathing [47]. A total of 37 studies (1543 patients) were included. The interstudy sensitivity heterogeneity was high (*I*_2_ = 83%; *p* < 0.001), with a wide percentage error (47%), implying that completely noninvasive CO devices are not interchangeable with thermodilution.

In terms of fluid responsiveness, bioreactance-based devices have been studied in both spontaneously breathing and mechanically ventilated patients with shock in combination with the passive leg raise test (PLR). Specifically, NICOM has been validated in the assessment of fluid responsiveness by PLR [48–50]. Benomar et al. used the bioreactance-based PLR test to study fluid responsiveness in 75 ICU patients after cardiac surgery and estimated the precision of NICOM to derive the least minimum significant change in CO of 8.85% [48]. Bioreactance-based PLR had a sensitivity of 88% and 100% specificity for predicting fluid responsiveness. On ROC curve analysis, the area under the curve was 0.84 to detect fluid responsiveness >10%. Galarza et al. showed in 32 critically ill patients that bioreactance-based detection of >10% increase in cardiac index with PLR had a sensitivity of 92% (95% CI 62–91%) and specificity of 80% (95% CI 56–94%); area under the curve was 088 [51]. However, the percentage error of the Starling-SV when compared with transpulmonary thermodilution was more than 30%, suggesting that the two methodologies may not be interchangeable when it comes to CO measurements. In a study among hemodynamically unstable ICU patients using carotid and brachial arterial Doppler ultrasound flow as the reference technique, almost 100% concordance was found between fluid responsiveness determined by carotid flow and the NICOM system. This study reported a sensitivity of 94% and specificity of 86% for the use of bioreactance to determine fluid responsiveness [49]. NICOM has been compared with esophageal Doppler monitoring (EDM) in measuring fluid responsiveness among patients undergoing major surgery [52, 53]. Waldron et al. found that the agreement between monitors was 60% and 66% at 5 and 15 minutes, respectively, using a 10% increase in SV after fluid challenge, with no systematic disagreement at any point, while Pascale et al. demonstrated a higher accuracy for predicting fluid responsiveness of about 80% (sensitivity 80%; specificity 82.6%) with NICOM relative to EDM. Chopra et al. showed that the percent change in SV index measured by NICOM after a PLR has a precision of ±9% (standard deviation) in both critically ill patients and healthy volunteers [54]. Stroke volume variability from NICOM has been demonstrated to successfully predict fluid responsiveness in patients requiring a prone positioning [55].

First-version NICOM provided CO readings averaged over 30 seconds, creating concerns in its ability to track very rapid changes in SV (pertinent in the assessment of fluid responsiveness). Alteration of the algorithm has led to the Starling-SV device for which the averaging time of CO has been reduced to 8 seconds. Bioreactance-based measurements are limited in situations where there is no association between aortic systolic deformation and SV (e.g., aortic dissection or aortic prosthesis), severe anemia, pulmonary arterial hypertension, or due to physical limitations such as obesity and large pleural effusions. Pitfalls and limitations of using bioreactance-based noninvasive cardiac output monitoring in the critical care unit and in intraoperative settings are shown in supplementary material Table 2 [25, 42, 48, 52, 53, 56–58].

On the basis of the preceding discussion, alternative approaches towards hemodynamic monitoring in SAH can involve minimally invasive methods for the continuous monitoring of CI and fluid responsiveness [12]. Such strategies in combination with early goal-directed therapy have recently been shown to hold promise in reducing the incidence of DCI and improving functional outcome at 3 months compared with standard postoperative fluid management, especially in patients with poor WFNS grades [13, 59, 60]. Among the tenets of care for all patients with SAH is the maintenance of euvolemia and hemodynamic optimization with the ultimate goal of preventing, or limiting, DCI. In addition, a nontrivial percentage of patients with SAH may concurrently suffer from neurogenic stress cardiomyopathy of various degrees of severity. For these reasons, the ability to noninvasively and continuously monitor hemodynamics at the bedside, as early on as from the emergency room, is an important advance in the care of these patients. The NICOM device offers the opportunity for monitoring more patients, and earlier on. It can serve as a screening tool for selected patients who may require further invasive hemodynamic monitoring as indicated. Furthermore, by monitoring fluid responsiveness, it offers the opportunity for rationalizing fluid management with the goal of avoiding both hypovolemia and fluid overload.

In this manuscript, we reviewed the use of a noninvasive device that has been reported, in different ICU populations, to have an acceptable performance in terms of estimations and tracking of CI and fluid responsiveness. Additional considerations include the ability to monitor patients without central or arterial lines, the user-friendly nature of the device, and the ability to employ it in non-ICU settings. In terms of our limited evaluation of this technology, we found CI and fluid responsiveness estimations consistent and expected as compared to the overall normal cardiac function (by TTE and clinical course) demonstrated by our patients with mostly low-grade SAH. The idea of monitoring fluid responsiveness in order to rationalize fluid management in patients with SAH is promising and should be further explored. However, this short report precludes any conclusions in terms of device performance in patients with high-grade SAH or patients with stress cardiomyopathy or under active hemodynamic augmentation with vasoactive medications. Future work is planned to investigate the role of such monitoring in “hemodynamically active” SAH patients.

## 3. Conclusions

Estimation and manipulation of effective circulating volume, cardiac function, and stroke volume are central features in the critical care approach to patients with SAH. Reliance on static preload markers, fluid balances, and indiscriminate fluid loading should be discouraged in view of their unreliability and potential harm in terms of both systemic and neurologic adverse effects. Instead, accumulating evidence across critically ill populations suggests that hemodynamic optimization can be better informed by direct measurements of compartmental volumes, continuous assessment of stroke volume and cardiac performance, and testing of fluid responsiveness. A number of minimally invasive technologies can provide such monitoring; our aim, here, was to review a noninvasive bioreactance-based technique and comment on our preliminary experience as tested in a pilot cohort of SAH patients.

## Data Availability

The patient clinical data are securely stored with the authors, and the hemodynamic data were extracted by the machine and kept for one year after the study.

## Supplementary Materials

Supplementary Materials Supplementary material Table 1: patient characteristics; supplementary material Table 2: pitfalls and limitations of bioreactance-based noninvasive cardiac output monitoring; and supplementary material Table 3: cardiac index, stroke volume, and fluid responsiveness.
