# Supplementary material for: Bioreactance-Based Noninvasive Fluid Responsiveness and Cardiac Output Monitoring: A Pilot Study in Patients with Aneurysmal Subarachnoid Hemorrhage and Literature Review
Source: Crit Care Res Pract. 2020 Sep 15;2020:2748181. doi: 10.1155/2020/2748181 (PMC7512079; doi:10.1155/2020/2748181)
Supplement: Supplementary Materials — Supplementary material Table 1: patient characteristics; supplementary material Table 2: pitfalls and limitations of bioreactance-based noninvasive cardiac output monitoring; and supplementary material Table 3: cardiac index, stroke volume, and fluid responsiveness. [file 2748181.f1.zip › 2748181.f1/Supplementary material Table 1.docx]

**Supplementary material Table 1. Patient characteristics (N = 10)**

| Variable | N [median (IQR)] |
| --- | --- |
| Age | [57 (43-71)] |
| Gender (female) | 6 |
| APACHE II | [5.5 (5-10)] |
| Admission Glasgow Coma Scale | [15 (12-15)] |
| WFNS Grade  1  2  3  4  5 | [1 (1-3)]  6  1  1  1  1 |
| Modified Fischer Scale  1  2  3  4 | [3.5 (1-4)]  3  1  1  5 |
| **Medical History**  Hypertension  Congestive Heart Failure  Diabetes Mellitus | 9  0  3 |
| Radiographic Vasospasm | 1 |
| Delayed Cerebral Ischemia | 2 |
| ICU LOS in days | [12 (11-12)] |
| Modified Rankin Scale at discharge  0  1  2  3  4  5  6 | [3 (1-4)]  2  2  1  0  5  0  0 |

Abbreviations: APACHE II: acute physiology and chronic health evaluation II; WFNS: world federation of neurological surgeons; LOS: length of stay; IQR; interquartile range.
