# Supplementary material for: Bioreactance-Based Noninvasive Fluid Responsiveness and Cardiac Output Monitoring: A Pilot Study in Patients with Aneurysmal Subarachnoid Hemorrhage and Literature Review
Source: Crit Care Res Pract. 2020 Sep 15;2020:2748181. doi: 10.1155/2020/2748181 (PMC7512079; doi:10.1155/2020/2748181)
Supplement: Supplementary Materials — Supplementary material Table 1: patient characteristics; supplementary material Table 2: pitfalls and limitations of bioreactance-based noninvasive cardiac output monitoring; and supplementary material Table 3: cardiac index, stroke volume, and fluid responsiveness. [file 2748181.f1.zip › 2748181.f1/Supplementary material Table 2.docx]

**Supplementary material Table 2.**

Pitfalls and Limitations of Bioreactance based Noninvasive Cardiac Output Monitoring

| Critical Care unit | Intraoperative Monitoring |
| --- | --- |
| Bioreactance technique is dependent on diffusion of electrical current. Thus, interstitial edema may interfere with measurements  Non-fluid interventions (e.g., vasopressors or postural change) can have an effect on bioreactance based CO measurements. A possible influence of current flux pathways related to the position of the lower chest NICOM electrodes could cause these effects.   - The mathematical model for bioreactance is based on the assumption that blood resistivity is a constant. Since blood resistivity is proportional to hematocrit, significant hemodilution in critically ill patients can skew bioreactance CO measurement. This limitation is not significant for hematocrit values ranging from 25% to 45%. - The hemodynamic effects of PLR reach their maximum within 1 min, diminishing rapidly thereafter in some patients, especially in patients with severe sepsis and capillary leak.^42^ - Some studies show that the definition of a fluid responder (an increase in SV and/or CO of at least 10% after a fluid bolus of 500 mL 0.9% NaCl) might not be suitable in untreated patients with sepsis. More fluid, at least 1000 ml, was needed to induce a significant response.^48,52,56^   Bioreactance device with a longer averaging period of >30 seconds can yield inaccurate measurements of the hemodynamic effects induced by PLR test, in critically ill patients. | Some studies demonstrate than NICOM has limited reliability when used for intraoperative monitoring particularly in patients receiving abdominal surgery.  In patients undergoing abdominal surgery, agreement between NICOM and EDM in assessing response to a fluid challenge for guiding goal-directed fluid therapy was poor (60- 66%).^52^   - A bias of −6.9 ml, wide limits of agreement (−22.9 to 36.8 ml), was demonstrated between EDM and NICOM when used during major open-abdominal surgery.^57^ - Bioreactance based CO measurements are less reliable during electrical diathermy. Cautery interference can be significant.^53,57^ - Trending ability is less reliable and significantly influenced during upper abdominal interventions (e.g., open retractor placement, laparoscopic insufflation, and head-down tilt), with unpredictable shifts in CO >1 l/min/m.^31,58^   Changes in geometry of the upper abdomen related to the interventions may affect the bioreactance readings. Reliability of NICOM-derived SV according to patient position change or any surgical incision between the upper thoracic and the lower thoracic NICOM electrodes has not been clarified.  NICOM calibration shifts during specific phases of the surgical intervention is a potential determinant of the suboptimal concordance between NICOM and other modalities used to measure CO and fluid responsiveness. |

Abbreviations:

CO cardiac output; SV stroke volume; PLR Passive Leg Raise; EDM Esophageal Doppler Monitoring; NICOM Noninvasive Cardiac Output Monitor.
