# Supplementary material for: Bioreactance-Based Noninvasive Fluid Responsiveness and Cardiac Output Monitoring: A Pilot Study in Patients with Aneurysmal Subarachnoid Hemorrhage and Literature Review
Source: Crit Care Res Pract. 2020 Sep 15;2020:2748181. doi: 10.1155/2020/2748181 (PMC7512079; doi:10.1155/2020/2748181)
Supplement: Supplementary Materials — Supplementary material Table 1: patient characteristics; supplementary material Table 2: pitfalls and limitations of bioreactance-based noninvasive cardiac output monitoring; and supplementary material Table 3: cardiac index, stroke volume, and fluid responsiveness. [file 2748181.f1.zip › 2748181.f1/Supplementary Material_table 3_R2.docx]

**Supplementary material. Table 3. Cardiac index, stroke volume and fluid responsiveness**

| **Subject** | **Summary Statistics** | **CI** | **SV** | **% Change in SV induced by PLR*** |
| --- | --- | --- | --- | --- |
| 1 | Average | 3.06 | 94.96 | 19.16 |
|  | Standard Deviation | 0.66 | 14.95 | 8.07 |
|  | Median | 3.05 | 94.49 | 16.65 |
|  | Minimum | 1.43 | 49.11 | 11.30 |
|  | Maximum | 5.87 | 149.30 | 33.10 |
| 2 | Average | 3.13 | 69.03 | 17.34 |
|  | Standard Deviation | 0.73 | 11.94 | 9.59 |
|  | Median | 3.10 | 68.98 | 17.90 |
|  | Minimum | 1.44 | 36.32 | 1.20 |
|  | Maximum | 5.92 | 128.87 | 38.40 |
| 3 | Average | 3.68 | 98.32 | 19.93 |
|  | Standard Deviation | 0.72 | 16.08 | 12.28 |
|  | Median | 3.65 | 99.32 | 15.05 |
|  | Minimum | 1.90 | 46.61 | 10.10 |
|  | Maximum | 6.67 | 154.93 | 42.00 |
| 4 | Average | 3.56 | 101.32 | 23.89 |
|  | Standard Deviation | 1.18 | 30.44 | 12.98 |
|  | Median | 3.38 | 98.72 | 21.00 |
|  | Minimum | 0.60 | 19.71 | 10.10 |
|  | Maximum | 10.92 | 281.80 | 51.30 |
| 5 | Average | 3.31 | 61.17 | 23.63 |
|  | Standard Deviation | 0.71 | 14.08 | 17.58 |
|  | Median | 3.39 | 63.86 | 15.95 |
|  | Minimum | 1.37 | 25.51 | 11.30 |
|  | Maximum | 5.54 | 99.48 | 57.00 |
| 6 | Average | 2.60 | 56.65 | 18.57 |
|  | Standard Deviation | 0.76 | 15.27 | 7.48 |
|  | Median | 2.66 | 59.01 | 15.50 |
|  | Minimum | 0.54 | 13.17 | 11.70 |
|  | Maximum | 5.06 | 104.65 | 32.60 |
| 7 | Average | 3.35 | 69.44 | 29.26 |
|  | Standard Deviation | 0.90 | 17.33 | 14.01 |
|  | Median | 3.35 | 70.04 | 32.50 |
|  | Minimum | 0.90 | 22.57 | 11.20 |
|  | Maximum | 6.26 | 130.75 | 50.30 |
| 8 | Average | 3.71 | 97.19 | 28.04 |
|  | Standard Deviation | 0.72 | 20.92 | 17.45 |
|  | Median | 3.66 | 96.07 | 20.85 |
|  | Minimum | 2.09 | 47.94 | 11.50 |
|  | Maximum | 7.37 | 182.10 | 72.00 |
| 9 | Average | 3.01 | 64.05 | 16.70 |
|  | Standard Deviation | 0.73 | 11.44 | 11.03 |
|  | Median | 2.91 | 62.85 | 13.30 |
|  | Minimum | 0.97 | 17.53 | 7.70 |
|  | Maximum | 6.37 | 113.85 | 32.50 |
| 10 | Average | 2.79 | 65.32 | 21.93 |
|  | Standard Deviation | 0.64 | 10.54 | 9.02 |
|  | Median | 2.66 | 64.39 | 20.20 |
|  | Minimum | 1.35 | 32.96 | 12.50 |
|  | Maximum | 10.28 | 223.85 | 43.50 |

* <10% implies not fluid responsive.

Abbreviations: CI: cardiac index; SV: stroke volume; PLR: passive leg raise.
